# Measles in the Post-COVID Era: Incidence Trends, Vaccination Coverage, Demographic and Subnational Distribution in Saudi Arabia, 2015–2024

**DOI:** 10.3390/vaccines14050445

**Published:** 2026-05-16

**Authors:** Lama Alzamil

**Affiliations:** Department of Clinical Laboratories Sciences, College of Applied Medical Sciences, King Saud University, Riyadh 12372, Saudi Arabia; lalzamil@ksu.edu.sa

**Keywords:** measles, COVID-19 pandemic, vaccination coverage, Saudi Arabia, Eastern Mediterranean Region, Gulf Cooperation Council, immunisation disruption, post-pandemic rebound, mass gatherings

## Abstract

**Background/Objectives:** The COVID-19 pandemic disrupted routine immunisation globally. Saudi Arabia presents a unique epidemiological context for measles, combining high vaccination coverage with mass pilgrimages and a large expatriate workforce. This study examined measles incidence trends, vaccination coverage, and demographic and geographic burden distribution in Saudi Arabia (2015–2024), with comparative analysis against GCC countries, the Eastern Mediterranean Region (EMR), and global data. **Methods**: Annual incidence and vaccination coverage data were obtained from the WHO Global Health Observatory and WHO/UNICEF WUENIC; monthly, regional, age- and nationality-stratified data from the Saudi Ministry of Health Annual Statistical Book (2015–2024). Incidence was expressed per 1,000,000 population across three epochs: pre-COVID-19 (2015–2019), pandemic disruption (2020–2021), and post-COVID-19 rebound (2022–2024). Descriptive analyses included period means, percentage changes, rate ratios, and rate differences. **Results**: Pre-COVID-19 incidence (mean 19.7/1,000,000) remained below EMR and global averages. The pandemic produced near-complete suppression (−96.6% to 1.1/1,000,000 in 2020), exceeding global (−82.2%) and EMR (−61.2%) declines. A marked rebound occurred in 2023 (67.8/1,000,000), surpassing the pre-pandemic peak despite MCV1/MCV2 coverage above 96%. Non-Saudi nationals bore disproportionate burden in 2021 (20.7 vs. 1.1/1,000,000) and 2023 (70.4 vs. 64.8/1,000,000). Children under 15 accounted for 71.6–90.6% of annual cases, with the 5–<15-year group’s contribution rising from 12.7% (pre-COVID mean) to 27.7% in 2024. Geographic burden shifted annually with no consistently dominant region. **Conclusions**: Saudi Arabia’s post-pandemic rebound despite high national coverage implicates sub-population susceptibility gaps among non-national residents and school-age children, alongside importation risks from mass pilgrimage. Targeted strategies addressing demographic and geographic heterogeneity are essential to meet WHO 2030 elimination targets.

## 1. Introduction

Measles remains one of the most contagious vaccine-preventable diseases and a leading cause of childhood mortality worldwide, despite the availability of a safe and highly effective vaccine for over six decades [1]. Global vaccination programmes have achieved remarkable progress, with estimated measles deaths falling by more than 80% between 2000 and 2016, and the number of countries achieving measles elimination increased substantially during the same period [2]. Nevertheless, this progress has proven fragile. Approximately 10.3 million measles infections and an estimated 107,500 deaths were recorded in 2023, with the majority being children under five, representing a near-doubling of the global burden since 2021 [2]. The WHO 2030 measles elimination targets, to which all six regional offices are committed, remain at serious risk [3].

Measles transmission is sustained when population immunity falls below the herd immunity threshold, with varying R0 estimates reported [4]. A two-dose schedule of measles-containing vaccine (MCV), introduced in many national programmes from 1989 onwards, provides greater than 97% efficacy when both doses are administered [5]. However, even modest and geographically clustered shortfalls in coverage, particularly among mobile, migrant, or marginalised sub-populations, are sufficient to sustain transmission chains and spark outbreaks, as demonstrated in New York (2018–2019), Madagascar (2018–2019), the Philippines (2016–2019), and the Democratic Republic of Congo (2018–2020) [6,7,8,9].

The COVID-19 pandemic introduced an acute global disruption to routine immunisation services. Physical distancing measures, facility closures, caregiver reluctance to attend health facilities, and redeployment of health workers collectively caused an estimated 25 million children worldwide to miss scheduled measles doses in 2021 alone, the largest single-year increase in unvaccinated children in at least three decades [10]. Modelling studies confirmed that measles vaccination disruptions of even 2–4 months substantially increase the probability of subsequent outbreaks [11]. The Eastern Mediterranean Region (EMR), a WHO region comprising 22 member states stretching from Morocco to Pakistan, has carried a disproportionate share of the global measles burden throughout the past decade, reflecting the region’s combination of high-burden conflict-affected states, large mobile populations, and variable immunisation infrastructure [12]. The COVID-19 pandemic exacerbated this burden considerably, with annual regional measles incidence increasing between 2019 and 2022 [13].

Saudi Arabia, an EMR member state with one of the region’s most established national immunisation programmes, introduced mandatory measles vaccination in 1982, resulting in an increase in coverage from approximately 8% in 1980 to over 90% by 1990, and subsequently adopted a two-dose schedule in 1991 [14]. Despite sustained MCV1 coverage, Saudi Arabia experienced a 2023 rebound that more than doubled its pre-pandemic peak [15]. Understanding this paradox requires examining structural vulnerabilities in Saudi Arabia’s epidemiological profile that aggregate metrics do not capture. First, Saudi Arabia hosts the two largest annual mass gatherings in the world: Hajj pilgrimage, which draws approximately 1.8–2.5 million international visitors each year [16], and Umrah, which attracted over 13.5 million pilgrims in 2022–2023 [17]. Both events bring together individuals from countries with widely varying immunisation histories, creating concentrated importation risk and dense transmission contexts. Second, approximately 42% of Saudi Arabia’s total population consists of non-national residents [18], predominantly labour migrants from South Asia, Southeast Asia, and Sub-Saharan Africa [19], regions where measles burden and vaccination coverage vary considerably. This population is often excluded from routine national immunisation registers and may not be fully captured in WHO/UNICEF Estimates of National Immunization Coverage (WUENIC) estimates, creating a documented discrepancy between reported national coverage and actual population immunity. Third, Saudi Arabia shares a lengthy southern border with Yemen, which has experienced one of the largest sustained measles outbreaks globally throughout 2018–2024, driven by war-related health system collapse and displacement [20].

Despite the recognised importance of Saudi Arabia’s post-pandemic measles trajectory, the literature lacks a systematic analysis that integrates national incidence trends, regional comparisons, subnational burden characterisation, and demographic disaggregation across the full decade. Prior studies have examined COVID-19’s impact on vaccination coverage within specific Saudi regions [21,22] or characterised individual outbreaks [15], but none has applied this scope of analysis across the 2015–2024 period. Understanding whether the post-pandemic rebound reflects a systemic immunity gap or localised importation dynamics is essential for prioritising intervention strategies. This study aims to compare measles incidence trends across three epidemiological periods: pre-COVID-19 (2015–2019), pandemic disruption (2020–2021), and post-COVID-19 rebound (2022–2024), in Saudi Arabia relative to EMR, global, and GCC benchmarks; to assess changes in MCV1 and MCV2 coverage before and after the COVID-19 pandemic; to characterise the subnational geographic distribution of measles burden across Saudi Arabia’s administrative regions over the full study period; to examine the monthly distribution of cases across 2015–2024; to analyse nationality-stratified incidence among Saudi and non-Saudi populations; and to describe the age distribution of cases across the decade.

## 2. Materials and Methods

Annual measles reported cases and incidence rates for Saudi Arabia, the Eastern Mediterranean Region (EMR), globally, and for Gulf Cooperation Council (GCC) member states (Bahrain, Kuwait, Oman, Qatar, and the United Arab Emirates) were retrieved from the WHO Global Health Observatory (GHO) surveillance database [23]. Vaccination coverage data for first-dose (MCV1) and second-dose (MCV2) measles-containing vaccines were extracted from the WHO/UNICEF Estimates of National Immunization Coverage (WUENIC) [24]. Annual measles case counts disaggregated by month, administrative region, age group, and nationality were obtained from the Saudi Ministry of Health Annual Statistical Book for the years 2015–2024 [25]. Regional case counts were aggregated to Saudi Arabia’s 13 administrative regions and matched with GASTAT annual mid-year population estimates disaggregated by nationality to calculate region-specific attack rates and nationality-stratified incidence rates. GASTAT census denominators from the 2022 National Census [18] were used exclusively for Table 3 subnational attack rates. Population estimates for 2023 and 2024 are provisional GASTAT figures.

The study period was stratified into three epidemiological epochs: pre-COVID-19 baseline (2015–2019), COVID-19 disruption (2020–2021), and post-COVID-19 rebound (2022–2024). This stratification was informed by documented global immunisation service disruptions during 2020–2021 and the subsequent post-pandemic resurgence reported across multiple WHO regions [11]. Measles incidence rates were expressed as cases per 1,000,000 population per year, consistent with WHO immunization portal reporting standards. Subnational attack rates were expressed as cases per 100,000 population using GASTAT census denominators, as described above. Trends over time were summarised descriptively by period, with period means calculated as simple averages of annual values within each epoch. The percentage change in incidence between periods and between specific years was calculated as: ((value_2_ − value_1_)/value_1_) × 100. Rate ratios (RRs) were computed by dividing Saudi Arabia’s annual incidence by the corresponding global or EMR incidence; rate differences (RDs) were calculated as the absolute difference between Saudi Arabia and the comparator incidence. Nationality-stratified incidence rates were calculated separately for Saudi and non-Saudi nationals using GASTAT mid-year population estimates disaggregated by nationality for each year. Age-stratified case distributions were expressed as the percentage share of annual national cases within each age group (<1, 1–<5, 5–<15, 15–<45, and ≥45 years). Monthly case distributions were examined across the full 2015–2024 study period to assess temporal transmission patterns. Vaccine coverage comparisons were assessed descriptively using period means and percentage point changes. All calculations were performed using Microsoft Excel and GraphPad Prism (version 11).

## 3. Results

### 3.1. Trends in Measles Incidence Pre- and Post-COVID-19

**Figure 1:**
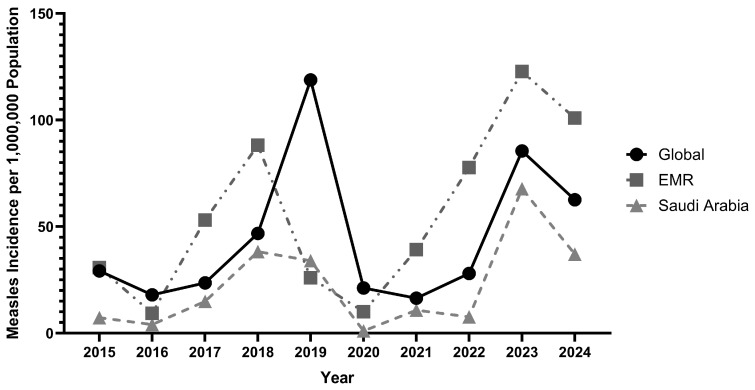
Measles incidence per 1,000,000 population in Saudi Arabia, the Eastern Mediterranean Region (EMR), and globally from 2015 to 2024. Data represent WHO-reported confirmed measles cases standardised to population size. EMR = Eastern Mediterranean Region.

During the pre-COVID-19 period (2015–2019), global measles incidence fluctuated substantially, rising from 29.2 per 1,000,000 in 2015 to a major peak of 118.8 per 1,000,000 in 2019, driven by large-scale outbreaks across multiple WHO regions (Figure 1; Table 1). The EMR followed a different trajectory, peaking earlier in 2018 (88.2 per 1,000,000) before declining sharply to 26.0 per 1,000,000 in 2019. Saudi Arabia’s pre-COVID incidence was consistently below both global and EMR rates, with a mean of 19.7 per 1,000,000 over 2015–2019, peaking at 38.3 per 1,000,000 in 2018. Uniquely, in 2019, Saudi Arabia’s incidence (34.0 per 1,000,000) exceeded the EMR regional average (26.0 per 1,000,000).

The COVID-19 pandemic disruption period (2020–2021) produced sharp declines across all three comparators. Global incidence fell from 118.8 per 1,000,000 in 2019 to 21.2 per 1,000,000 in 2020, while EMR incidence declined to 10.1 per 1,000,000. Saudi Arabia experienced the most pronounced suppression, with incidence falling to 1.1 per 1,000,000 in 2020, the lowest recorded in the study period (Figure 1; Table 1).

From 2022 onwards, measles incidence rebounded across all comparators, peaking in 2023 (Figure 1; Table 1). Saudi Arabia’s rebound was proportionally the most pronounced: national incidence reached 67.8 per 1,000,000 in 2023, compared to a pre-pandemic maximum of 38.3 per 1,000,000 in 2018 and a pre-COVID period mean of 19.7 per 1,000,000. The EMR’s 2023 peak of 122.8 per 1,000,000 was the highest in the study period, exceeding its previous 2018 maximum of 88.2 per 1,000,000. By 2024, incidence declined in all three comparators, globally to 62.6 per 1,000,000, in Saudi Arabia to 37.1 per 1,000,000, and in the EMR to 100.9 per 1,000,000, indicating early recovery across the region.

Rate ratios (RR) and rate differences (RD) comparing Saudi Arabia to global incidence showed that Saudi Arabia maintained lower incidence throughout the study period, though the magnitude of this difference varied across periods (Table 1). During 2015–2019, the RR ranged from 0.228 to 0.818, indicating Saudi incidence was consistently below the global rate. In 2020, the RR fell to its lowest point of 0.052, reflecting near-complete suppression of transmission. The RR reached its post-pandemic peak of 0.793 in 2023, before declining to 0.593 in 2024. The rate difference was most pronounced in 2019 (−84.8 per 1,000,000), where the global spike was not mirrored in Saudi Arabia, and narrowest in 2021 (−5.6 per 1,000,000), reflecting convergence of both trajectories at low incidence levels during peak pandemic suppression.

Within the GCC, Saudi Arabia recorded the highest measles incidence in 2023 at 67.8 per 1,000,000 (Table 1). UAE (45.5 per 1,000,000) and Qatar (42.6 per 1,000,000) recorded the next highest rates, while Oman (5.0), Bahrain (1.9), and Kuwait (1.2) reported substantially lower rates. Kuwait 2020 data were unavailable in the WHO surveillance dataset and are excluded from period comparisons for that year.

### 3.2. Measles-Containing Vaccine Coverage Before and After COVID-19

Saudi Arabia maintained high measles vaccine coverage throughout the study period, with MCV1 remaining stable at 97.0% across both periods and MCV2 increasing marginally from 96.2% to 96.6% (+0.40 pp) (Table 2). Globally, MCV1 coverage declined from a pre-COVID mean of 85.2% to 82.8% (−2.40 pp), while MCV2 increased from 67.8% to 73.0% (+5.20 pp). In the EMR, MCV1 declined from 81.4% to 80.8% (−0.60 pp), with MCV2 increasing from 72.4% to 74.6% (+2.20 pp).

Within the GCC, MCV1 coverage was uniformly high and broadly comparable to Saudi Arabia’s 97.0% across both periods, ranging from 97.2% to 99.0% pre-COVID and 97.0% to 99.0% post-COVID (Table 2). Changes were minimal, ranging from 0.00 pp (Bahrain) to −1.80 pp (Qatar). MCV2 coverage showed greater variability relative to Saudi Arabia’s stable 96.2–96.6% range (Table 2). Bahrain and Oman maintained higher MCV2 coverage than Saudi Arabia in both periods (99.0% and 98.8% respectively), with no change across periods. Kuwait and UAE recorded declines of −3.00 pp and −3.60 pp respectively, falling below Saudi Arabia’s post-COVID MCV2 level by the end of the study period. Qatar recorded the largest MCV2 increase in the GCC (+3.00 pp), rising from 93.8% pre-COVID to 96.8% post-COVID, marginally exceeding Saudi Arabia’s post-COVID level.

### 3.3. Seasonal Distribution of Measles Cases in Saudi Arabia, 2015–2024

**Figure 2:**
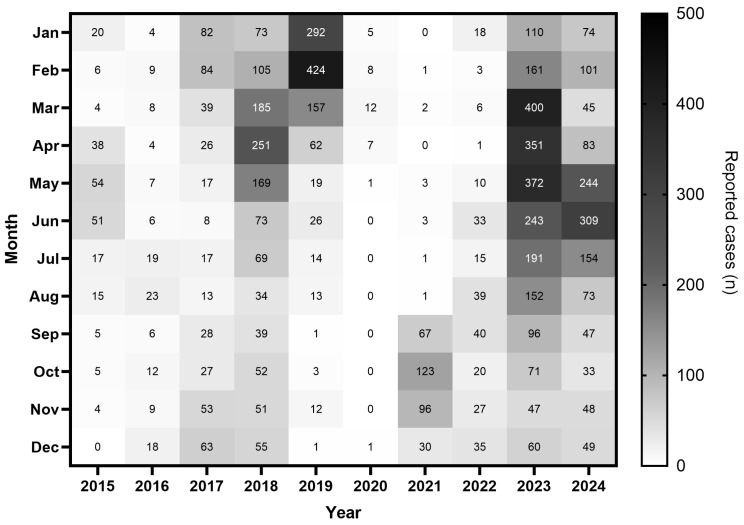
Monthly distribution of reported measles cases in Saudi Arabia, 2015–2024. Colour intensity reflects the absolute number of reported cases per cell, with darker shading indicating higher case counts (scale: 0–500). The 2019 peak was concentrated in the early months of the year, particularly January–February (292 and 424 cases, respectively). A near-complete suppression of cases is evident in 2020, consistent with pandemic-related reductions in transmission and surveillance activity. The post-COVID-19 rebound from 2023 onwards shows a broader seasonal distribution, with elevated case counts persisting across spring and summer months.

The monthly distribution of cases across 2015–2024 revealed a seasonal pattern that varied by epidemiological period (Figure 2). During the pre-COVID-19 period (2015–2019), case counts were highest in 2018 and 2019. In 2018, cases peaked in April (*n* = 251) and March (*n* = 185), while in 2019 the peak shifted earlier, with the highest counts recorded in February (*n* = 424) and January (*n* = 292).

The COVID-19 disruption period (2020–2021) was characterised by near-complete suppression in 2020, with zero or single-digit counts across most months, followed by a partial rebound in 2021 concentrated in the final quarter (September n = 67, October n = 123, November n = 96).

During the post-COVID-19 rebound (2022–2024), the 2023 peak was the most pronounced, with the highest monthly counts recorded in March (n = 400), May (n = 372), and April (n = 351), together accounting for 1123 cases. In 2024, elevated counts persisted through the first half of the year, particularly in June (n = 309) and May (n = 244).

### 3.4. Regional Distribution of Measles Cases in Saudi Arabia, 2015–2024

**Figure 3:**
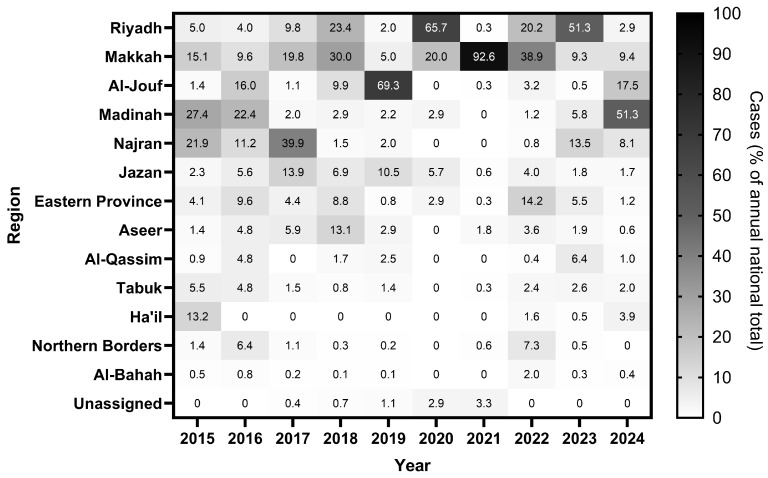
Regional distribution of measles cases as a percentage of the annual national total, Saudi Arabia, 2015–2024. Colour intensity reflects each region’s proportional contribution to the national case count in a given year, with darker shading indicating a higher percentage. Dominant regional contributions shift across epidemiological periods: Madinah and Najran contributed disproportionately in 2015–2016, Al-Jouf in 2019 (69.3%), Makkah in 2021 (92.6%), and Riyadh and Makkah during the post-COVID-19 rebound (2023). Values represent percentages of the annual national total; rows sum to 100% within each year.

The regional distribution of measles cases varied considerably across Saudi Arabia’s 13 administrative regions and epidemiological periods, with marked shifts in the dominant contributing regions over time (Figure 3).

During the pre-COVID-19 period (2015–2019), no single region consistently dominated. Madinah contributed the largest share in 2015 (27.4%) and 2016 (22.4%), while Najran was the dominant region in 2017 (39.9%). In 2018, Makkah (30.0%) and Aseer (13.1%) were the leading contributors. In 2019, Al-Jouf accounted for the majority of national cases (69.3%), representing the most geographically concentrated occurrence of the pre-pandemic period, with 717 of 1035 reported cases.

During the COVID-19 disruption period (2020–2021), Riyadh emerged as the dominant region in 2020 (65.7%), followed by Makkah in 2021 (92.6%; 313 of 338 national cases), the highest single-region contribution recorded across the entire study period (Figure 3).

During the post-COVID-19 rebound (2022–2024), the burden was more broadly distributed. In 2022, Makkah (38.9%) and Eastern Province (14.2%) were the leading regions. In 2023, Riyadh contributed the largest share (51.3%; 1157 of 2254 national cases). In 2024, Madinah emerged as the dominant region (51.3%; 647 of 1260 national cases). Ha’il, which recorded near-zero cases for most of the study period, showed a late increase in 2024 (49 cases; 3.9% of national total).

### 3.5. Age, Sex, and Nationality Distribution of Measles Cases in Saudi Arabia, 2015–2024

Across the study period, measles cases were reported among both Saudi nationals and non-Saudi nationals, with notable shifts in the relative burden between groups over time (Table 3). During the pre-COVID-19 period (2015–2019), Saudi nationals consistently accounted for the majority of cases, with incidence rates exceeding those of non-Saudi nationals in all years (Table 3). The largest absolute disparity was recorded in 2019, when Saudi national incidence reached 44.6 per 1,000,000 compared to 6.3 per 1,000,000 among non-Saudi nationals (Table 3). Sex distribution was broadly balanced across both nationality groups throughout the study period, with no consistent male or female predominance (Table 3).

**Table 3:** Reported measles cases and incidence rates per 1,000,000 population by nationality and sex, Saudi Arabia, 2015–2024.

Year	Saudi Nationals				Non-Saudi Nationals				Total Cases
	Male	Female	Total	Incidence Rate (per 1,000,000)	Male	Female	Total	Incidence Rate (per 1,000,000)	
**2015**	96	84	180	8.519	19	20	39	3.753	219
**2016**	55	42	97	4.834	13	15	28	2.398	125
**2017**	162	171	333	16.317	64	60	124	10.211	457
**2018**	445	429	874	42.083	144	138	282	22.301	1156
**2019**	480	461	941	44.590	40	43	83	6.329	1024
**2020**	7	10	17	0.793	11	6	17	1.252	34
**2021**	9	13	22	1.136	121	184	305	20.682	327
**2022**	61	60	121	6.439	54	72	126	9.415	247
**2023**	664	590	1254	64.806	511	489	1000	70.423	2254
**2024**	476	456	932	47.551	169	159	328	20.892	1260

Incidence rates represent the number of newly reported measles cases per 1,000,000 population per year, calculated using mid-year population estimates for Saudi nationals and non-Saudi nationals separately, obtained from the General Authority for Statistics (GASTAT), Kingdom of Saudi Arabia; estimates for 2023 and 2024 are provisional. Total cases are derived from the Ministry of Health Annual Statistical Book. Saudi and non-Saudi case counts may not sum exactly to the national total due to cases with nationality not recorded (unassigned cases: 2017 n = 2, 2018 n = 8, 2019 n = 11, 2020 *n* = 1, 2021 *n* = 11).

During the COVID-19 disruption period (2020–2021), the pattern reversed: non-Saudi nationals accounted for the majority of cases in 2021 (305 of 327; 93.3%), with an incidence of 20.7 per 1,000,000, substantially exceeding the Saudi national rate of 1.1 per 1,000,000 for that year (Table 3). During the post-COVID-19 rebound (2022–2024), incidence rose among both groups, though non-Saudi nationals recorded higher rates in 2023 (70.4 per 1,000,000) compared to Saudi nationals (64.8 per 1,000,000), the narrowest gap between the two groups outside the disruption period (Table 3).

**Figure 4:**
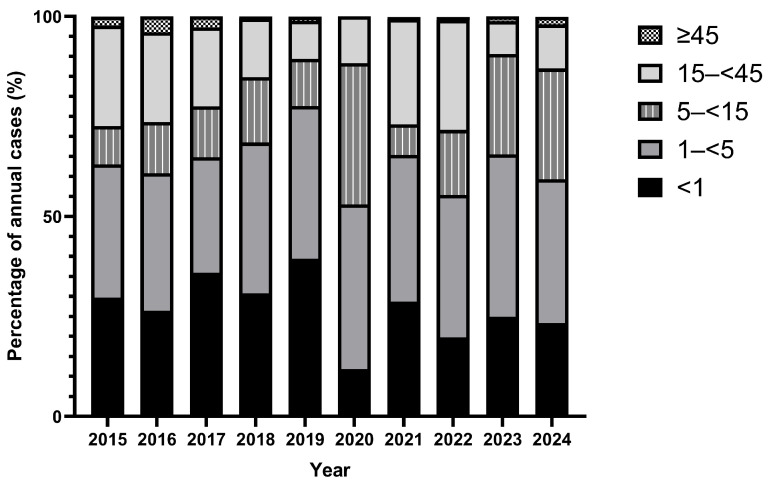
Age group distribution of reported measles cases as a percentage of annual total, Saudi Arabia, 2015–2024. Children under 15 years accounted for the majority of cases throughout the study period. The proportion of cases in the 5–<15 year age group increased notably in 2023 and 2024. 2020: Hajj pilgrimage suspended; total national cases = 34. Data source: Ministry of Health Annual Statistical Book, Saudi Arabia.

The age distribution of cases revealed a consistent pattern of disproportionate burden among children under five years across the study period (Figure 4). Children aged less than one year and those aged 1–<5 years together accounted for the majority of annual cases in most years, ranging from 44.8% in 2020 to 73.5% in 2019 (Figure 4). The 5–<15 age group contributed a notably higher proportion during the post-COVID-19 rebound, reaching 25.1% in 2023 (*n* = 565) and 27.7% in 2024 (n = 349), compared to a mean of 12.7% during the pre-COVID-19 period, suggesting disproportionate involvement of school-age children during the rebound (Figure 4). The 15–<45 and ≥45 age groups consistently contributed the smallest proportions, though the 15–<45 group showed a transient increase in 2020 (35.3%; *n* = 12) and 2022 (27.5%; *n* = 68) (Figure 4).

## 4. Discussion

This study examined the impact of the COVID-19 pandemic on measles incidence and vaccination coverage in Saudi Arabia over a ten-year period (2015–2024), with comparative analysis against the EMR, global benchmarks, and GCC countries. Saudi Arabia’s epidemiological trajectory across this period was defined by three distinct phases: a pre-COVID period of moderate but variable incidence (mean 19.7 per 1,000,000, 2015–2019), near-complete suppression during 2020–2021, and a pronounced post-pandemic rebound peaking at 67.8 per 1,000,000 in 2023 before partial recovery in 2024.

Saudi Arabia’s suppression of measles during 2020–2021 (Table 1) is consistent with the near-complete suspension of international pilgrimages [26]. The cancellation of Hajj for international pilgrims in 2020 and restriction to domestic pilgrims in 2021, substantially reduced the pressure that accompanies the arrival of millions of visitors from countries with varying immunisation histories. This is a unique feature of Saudi Arabia’s disease epidemiology with no equivalent in other EMR or GCC member states. In addition, COVID-19 containment measures (including border closures, movement restrictions, and reduced healthcare attendance) suppressed domestic transmission across all respiratory pathogens [27]. The combined effect contributed to a level of measles suppression that exceeded the global and regional average, an observation consistent with published evidence on the role of pandemic measures in reducing non-COVID infectious disease incidence in 2020 [11,28]. The resumption of international travel and pilgrimage from 2022 onward, combined with the accumulated immunity deficit from two years of suppressed natural exposure and documented disruptions to routine immunisation in some regions [21,22], created the conditions for the pronounced 2023 rebound. Although the EMR experienced a post-pandemic resurgence reaching 122.8 per 1,000,000 in 2023 (Table 1), Saudi Arabia’s increase relative to its own pre-pandemic peak was substantially larger. Regional comparisons must be interpreted with caution, as EMR trends are disproportionately shaped by conflict-affected countries such as Yemen and Somalia, where measles resurgence reflects health system collapse and mass displacement rather than vaccination failure [20,26]. In contrast, Saudi Arabia’s rebound occurred despite an intact health system and consistently high vaccination coverage (Table 2), indicating a different underlying mechanism. By 2024, incidence declined more rapidly in Saudi Arabia (−44.1%) than globally (−29.0%) or regionally (−17.8%), supporting the interpretation of a transient outbreak rather than a sustained shift in transmission dynamics (Table 1); this pattern has been observed in other high-coverage settings following pandemic disruption [28].

The central analytical challenge of this study is the apparent contradiction between Saudi Arabia’s sustained high vaccination coverage and its pronounced post-pandemic rebound. National MCV1 coverage of 97.0% (Table 2), well above the 95% herd immunity threshold for measles [4], was maintained throughout the study period, and second-dose coverage remained above 96%. At the national level, these figures should be sufficient to prevent large-scale transmission. Several structural features of Saudi Arabia’s demographic and epidemiological profile help explain this paradox. First, WHO/UNICEF WUENIC estimates are calculated from reports submitted by national immunisation programmes and may not fully capture vaccination status among non-national residents, who constitute 41.6% of the total registered population (Table 3) and are drawn predominantly from countries where measles burden and vaccination coverage vary considerably. If this sub-population is systematically under-vaccinated relative to the national average, the effective population immunity is lower than WUENIC estimates suggest [29]. Second, even with high average coverage, geographically or demographically clustered pockets of susceptibility, as demonstrated in pre-pandemic outbreaks in New York, Madagascar, and the Philippines [6,7,8], are sufficient to sustain measles transmission chains. Third, annual importation pressure through Hajj and Umrah creates a concentrated transmission context for susceptible sub-groups [15,30]. A fourth structural vulnerability not captured by MCV coverage metrics is the susceptibility window in infants below MCV1 eligibility age. In near-elimination settings maternal antibody levels are lower, resulting in earlier decay of passive immunity and a wider susceptibility gap before the first vaccine dose is administered [31]. This mechanism provides an explanatory basis for the consistently high infant case burden observed throughout this study (11.8–39.4% of annual cases in infants under one year).

Comparison with other GCC countries suggests that pilgrim-associated importation may contribute to the observed differences. Saudi Arabia’s 2023 incidence of 67.8 per 1,000,000 exceeded that of both UAE (45.5) and Qatar (42.6) (Table 1), despite these countries sharing comparable migrant labour dynamics and similar income levels [32]. Bahrain, Kuwait, and Oman reported substantially lower rates (≤5.0 per 1,000,000). It should be noted that Qatar’s smaller population size (approximately 3 million) renders its incidence estimate more susceptible to year-to-year variation from single outbreak events than Saudi Arabia’s population-adjusted rate; the UAE figure (approximately 10 million) is comparatively more stable as a benchmark. The three higher-rate GCC states share the characteristics of high international transit volumes and large migrant populations, while the lower-rate states have comparatively smaller international footprints. Critically, Kuwait recorded the largest MCV2 decline among lower-incidence GCC states (−3.00 pp, Table 2) yet reported one of the lowest 2023 rates in the group (1.2 per 1,000,000). UAE, despite the largest overall MCV2 decline (−3.60 pp), recorded the second-highest 2023 incidence (45.5 per 1,000,000), consistent with its high international transit volume. The absence of a consistent relationship between coverage decline and rebound severity within the GCC argues against vaccination programme disruption as the primary driver of Saudi Arabia’s rebound, and instead implicates importation dynamics specific to Saudi Arabia, particularly the scale of Hajj and Umrah [33], as the differentiating exposure. This is supported by mathematical modelling, which has shown that just a few infectious pilgrims traveling from countries with high measles rates can be enough to spark ongoing spread in the crowded conditions of Makkah [34].

Nationality-stratified analysis across the decade revealed a shift in the distribution of measles burden between Saudi and non-Saudi populations. During 2015–2019, Saudi nationals recorded higher incidence rates than non-Saudi nationals in every year, with Saudi nationals reaching 44.6 per 1,000,000 in 2019, nearly seven times the non-Saudi rate of 6.3 per 1,000,000. This pattern reversed from 2020 onward: non-Saudi rates exceeded Saudi rates in four consecutive years (2020–2023), most markedly in 2021 when the non-Saudi rate was 18 times higher (20.7 vs. 1.1 per 1,000,000), and again in 2023 when both groups experienced high rates but non-Saudi incidence remained higher (70.4 vs. 64.8 per 1,000,000). The 2021 finding is particularly notable: with Hajj restricted to approximately 60,000 domestic pilgrims, 305 of 327 national cases (93.3%) occurred among non-Saudis, suggesting a non-Saudi-specific exposure event, most plausibly reflecting clustering within migrant worker accommodation or disparate access to vaccination services during COVID-19 disruption. This is consistent with documented structural barriers to immunisation among migrants in the MENA region [29]. By 2024, the pattern reversed again, with Saudi rates exceeding non-Saudi rates (47.6 vs. 20.9 per 1,000,000), suggesting a return toward pre-pandemic transmission dynamics. These nationality-stratified patterns are not captured by aggregate WUENIC coverage estimates and underscore that effective population immunity in Saudi Arabia cannot be assessed without explicit consideration of the non-national population.

Examination of monthly case distribution across the full study period (Figure 2) reveals no consistent seasonal pattern. The month of peak transmission shifted markedly year to year: February (2017, 2019), April (2018), May (2015), June (2024), August (2016), September (2022), and October (2021). This inter-annual variability in peak timing is inconsistent with a fixed seasonal driver such as climate or a recurring calendar event, and instead reflects the stochastic dynamics of epidemic waves in a partially immune population, a pattern well documented in the measles literature, where subtle shifts in transmission conditions have been shown to produce spontaneous, unpredictable changes in epidemic periodicity [35]. The 2023 spring peak (March, 400 cases; 17.7% of annual total) represents a notable exception: the concentration of cases between March and May (49.8% of 2023 annual total) overlaps with the peak Umrah pilgrimage season and precedes Hajj [26]. However, projecting this pattern as a general seasonal rule is not supported by the broader decade of data.

Subnational analysis across the full decade reveals a striking instability in geographic burden that is obscured by aggregate national figures (Figure 3). The dominant region changed every year between 2015 and 2024: Madinah led in 2015 and 2016, Najran in 2017, Makkah in 2018, Al-Jouf in 2019, Riyadh in 2020, Makkah again in 2021 and 2022, Riyadh in 2023, and Madinah in 2024. Over the full decade, Riyadh (22.7%), Makkah (18.0%), and Al-Jouf (15.4%) accounted for the largest shares of cumulative burden, yet none was consistently dominant across years. This shifting pattern is consistent with epidemic wave dynamics driven by susceptible population accumulation in different regions at different times, rather than fixed geographic risk factors [36]. Two specific concentrations merit discussion. Al-Jouf’s disproportionate burden in 2019 is most plausibly explained by cross-border importation from neighbouring countries; Iraq in particular recorded 3619 confirmed measles cases that year [37], this is reinforced by the fact that the land border crossing between Saudi Arabia and Iraq, which was closed since 1990, was formally reopened in October 2019 [38]. Similarly, Najran’s disproportionate burden in 2017 is consistent with cross-border importation from Yemen, which shares Saudi Arabia’s southern border and was experiencing rapid measles escalation from 2017 onward [20]. Makkah’s concentration of 92.6% of national cases in 2021 (Figure 3), which is a year of severely restricted Hajj attendance limited to domestic pilgrims [26], points to localised transmission within the city’s resident population rather than international importation.

Age-stratified analysis across the decade confirms that children under 15 years consistently account for the majority of the disease burden, accounting for 71.6–90.6% of annual cases (Figure 4). Infants under one year represented 11.8–39.4% of cases annually, a sustained signal of inadequate herd immunity to protect age-ineligible children. The most epidemiologically significant age-related finding is the progressive expansion of the 5–<15-year age group’s contribution over the rebound period (Figure 4; Section 3.5), suggesting accumulation of susceptible school-age children who missed vaccination during the COVID-19 period, consistent with documented declines in routine immunisation during 2020–2021 [21,22,39,40]. If this cohort effect persists, future outbreaks in Saudi Arabia may disproportionately affect older children, a pattern observed in other high-coverage settings following immunisation disruptions [35,36]. Targeted school-based catch-up vaccination campaigns addressing the 5–15-year cohort have been reported in Saudi Arabia [41].

These findings have several implications for measles control policy in Saudi Arabia. The persistence of high national vaccination coverage alongside post-pandemic resurgence indicates that aggregate coverage metrics are insufficient, and that surveillance and immunisation strategies should target sub-populations whose vaccination status may not be fully represented in WUENIC estimates, particularly labour migrants and their dependants. Saudi Arabia is classified by WHO/EMRO as a pre-elimination country, and its policy commitment to elimination by 2028 under Saudi Vision 2030 is reflected in the nationwide measles and rubella vaccination campaign launched by the Ministry of Health in November 2024 [41]. Achieving this target will require targeted immunisation outreach among non-national residents, school-based catch-up for COVID-era under-vaccinated cohorts, and enhanced subnational surveillance to detect localised susceptibility gaps before they sustain transmission. In this context, Saudi Arabia’s post-pandemic rebound exemplifies the measles-as-tracer principle articulated in the WHO Immunization Agenda 2030 (IA2030), whereby measles case detection serves as an early warning signal of programmatic and health system gaps that aggregate coverage metrics alone cannot reveal [42]. The current Hajj and Umrah vaccination requirements mandate meningococcal (MenACWY), polio, and yellow fever vaccines, but the measles-containing vaccine remains advisory only. Given that these pilgrimages draw millions annually from countries with widely heterogeneous MCV coverage, this gap is epidemiologically significant; mathematical modelling has shown that even a small number of infectious pilgrims from high-burden countries is sufficient to initiate transmission in Makkah and Madinah [34], and measles has been identified as an active clinical risk in current pilgrimage disease surveillance [43]. The meningococcal mandate, introduced following the 2000–2001 Hajj outbreaks and associated with a sustained decline in pilgrimage-associated disease, offers a direct policy precedent for formally elevating MCV to an enforced pre-travel requirement [44].

This study has several limitations that should inform interpretation of the findings. The ecological design prevents causal inference; observed associations between demographic features and attack rates cannot establish individual-level risk factors [45]. Individual-level data including vaccination status and healthcare access remain unavailable, limiting the depth of risk factor analysis. Regional MCV1 and MCV2 coverage data disaggregated to the 13 administrative regions are not reported in publicly available WUENIC estimates, precluding direct assessment of subnational heterogeneity in vaccination coverage. The cross-border importation hypotheses for Al-Jouf (2019) and Najran (2017) cannot be confirmed without genotypic data linking circulating strains to specific source populations. Region-specific immunisation data remain limited in the published literature; a recent subnational study from Al-Baha, Saudi Arabia, examining MMR vaccination coverage and confirmed cases across 2020–2024, similarly found persistent laboratory-confirmed infections despite nominally high coverage, with rural residence and incomplete vaccination identified as key risk factors [46]. The absence of serological surveillance data represents a further limitation; population-level seroprevalence studies have demonstrated that post-pandemic immunity gaps are invisible to coverage-based metrics alone and can explain outbreak vulnerability in settings with nominally high vaccination coverage [47]. Despite these limitations, this study provides a comprehensive ten-year epidemiological assessment integrating national trend analysis, regional benchmarking, subnational burden characterisation, and demographic disaggregation by age and nationality, an analytical scope not previously applied to Saudi Arabia’s measles epidemiology across the COVID-19 pandemic period.

## 5. Conclusions

This study demonstrates that Saudi Arabia’s measles epidemiology over 2015–2024 was characterised by marked inter-annual variability, with near-complete suppression in 2020 followed by a pronounced rebound to 67.8 per 1,000,000 in 2023 despite MCV1 and MCV2 coverage consistently above 96%. The disproportionate burden among non-Saudi nationals in 2021 and 2023, the shifting geographic concentration of cases across regions throughout the decade, and the increasing contribution of school-age children in 2023–2024 suggest that susceptibility gaps exist beyond what national coverage metrics capture. These findings point to the potential value of targeted immunisation outreach to non-national residents, school-based catch-up vaccination for COVID-era under-vaccinated cohorts, and flexible subnational surveillance capacity. Sustained progress toward WHO measles elimination targets will likely require strategies that move beyond aggregate coverage monitoring to address sub-population and geographic heterogeneity.

## Figures and Tables

**Table 1:** Annual measles incidence per 1,000,000 population in Saudi Arabia, the Eastern Mediterranean Region (EMR), globally, and selected Gulf Cooperation Council (GCC) countries, 2015–2024, with rate ratios and rate differences.

Period	Year	Incidence per 1,000,000 Population						GCC Comparators (Incidence per 1,000,000)				
		Global	EMR	Saudi Arabia	RR Saudi Arabia vs. Global	RD Saudi Arabia vs. Global	RR Saudi Arabia vs. EMR	UAE	Bahrain	Kuwait	Oman	Qatar
**Pre-COVID-19 (2015–2019)**	2015	29.2	30.8	7.3	0.25	−21.9	0.237	40	0	4.7	0	7.4
	2016	18	9.3	4.1	0.228	−13.9	0.441	24.7	0	2	25.3	11.5
	2017	23.6	53.1	14.9	0.631	−8.7	0.281	13.1	0	4.3	2	3.3
	2018	46.8	88.2	38.3	0.818	−8.5	0.434	18.4	0	9.7	0	0.7
	2019	118.8	26	34	0.286	−84.8	1.308	19.9	2	2.7	0	1.8
	**Period mean**	**47.3**	**41.5**	**19.7**	**0.416**	**−27.6**	**0.475**	**23.2**	**—**	**—**	**—**	**—**
**COVID-19 Dis-ruption (2020–2021)**	2020	21.2	10.1	1.1	0.052	−20.1	0.109	5.3	0	NA	1.5	1.1
	2021	16.4	39.2	10.8	0.659	−5.6	0.276	3.6	0	0.7	0	0.4
	**Period mean**	**18.8**	**24.7**	**6**	**0.319**	**−12.8**	**0.243**	**4.5**	**—**	**—**	**—**	**—**
**Post-COVID-19 Rebound (2022–2024)**	2022	28	77.7	7.7	0.275	−20.3	0.099	9.6	11.7	1.5	1.1	0.3
	2023	85.5	122.8	67.8	0.793	−17.7	0.552	45.5	1.9	1.2	5	42.6
	2024	62.6	100.9	37.1	0.593	−25.5	0.368	50.7	1.2	1.4	2.5	18
	**Period mean**	**58.7**	**100.5**	**37.5**	**0.639**	**−21.2**	**0.373**	**35.3**	**—**	**—**	**—**	**—**

RR = rate ratio; RD = rate difference; EMR = Eastern Mediterranean Region; KSA = Kingdom of Saudi Arabia; GCC = Gulf Cooperation Council. All incidence rates expressed per 1,000,000 population. RR is computed for KSA relative to both global and EMR rates; RD is computed for KSA relative to global rates only. Period means calculated from annual values within each epoch for Saudi Arabia, EMR, and global comparators. UAE period means included as the largest GCC state with sufficient population stability for period averaging. GCC country-level period means are not calculated for Bahrain, Kuwait, Oman, and Qatar owing to high inter-annual variability and population-size sensitivity; annual figures are presented for contextual comparison (—denotes absent period mean). Kuwait 2020 data unavailable in WHO surveillance dataset. Oman 2016 incidence of 25.3 per 1,000,000 reflects a contained outbreak in a small population (≈4.4 million); single-year spikes in small-population states should be interpreted with caution.

**Table 2:** Pre- and post-COVID-19 measles-containing vaccine coverage in Saudi Arabia, GCC countries, EMR, and globally (WUENIC, 2015–2024).

Country/Region	Dose	Pre-COVID Mean (2015–2019)	Post-COVID Mean (2020–2024)	Change (pp)
Global	MCV1	85.2%	82.8%	−2.40 pp
	MCV2	67.8%	73.0%	+5.20 pp
EMR	MCV1	81.4%	80.8%	−0.60 pp
	MCV2	72.4%	74.6%	+2.20 pp
Saudi Arabia	MCV1	97.0%	97.0%	0.00 pp
	MCV2	96.2%	96.6%	+0.40 pp
UAE	MCV1	99.0%	98.4%	−0.60 pp
	MCV2	96.6%	93.0%	−3.60 pp
Bahrain	MCV1	99.0%	99.0%	0.00 pp
	MCV2	99.0%	99.0%	0.00 pp
Kuwait	MCV1	97.2%	97.0%	−0.20 pp
	MCV2	96.4%	93.4%	−3.00 pp
Oman	MCV1	99.0%	98.6%	−0.40 pp
	MCV2	98.8%	98.8%	0.00 pp
Qatar	MCV1	99.0%	97.2%	−1.80 pp
	MCV2	93.8%	96.8%	+3.00 pp

pp = percentage points. WUENIC = WHO/UNICEF Estimates of National Immunization Coverage. EMR = Eastern Mediterranean Region. GCC = Gulf Cooperation Council. Pre-COVID mean calculated from annual WUENIC estimates for 2015–2019; post-COVID mean calculated from 2020–2024.

## Data Availability

All data used in this study are publicly available. Annual measles incidence data and GCC country-level figures were obtained from the WHO Global Health Observatory: https://www.who.int/data/gho. Vaccination coverage estimates were sourced from WHO/UNICEF WUENIC: https://immunizationdata.who.int/. Monthly and regional measles case data for Saudi Arabia were obtained from the Saudi Ministry of Health open data platform: https://open.data.gov.sa/en/publishers/c43616c3-f3ac-41b9-bbad-040670bc396d. Population and demographic data were obtained from the General Authority for Statistics (GASTAT) 2022 National Census: https://www.stats.gov.sa/en/census2022. All data used in this study are publicly available. Annual measles incidence data and GCC country-level figures were obtained from the WHO Global Health Observatory: https://www.who.int/data/gho (accessed on 12 April 2026). Vaccination coverage estimates were sourced from WHO/UNICEF WUENIC: https://immunizationdata.who.int/ (accessed on 3 September 2025). Monthly and regional measles case data for Saudi Arabia were obtained from the Saudi Ministry of Health Annual Statistical Book: https://www.moh.gov.sa/en/Ministry/Statistics/book/Pages/default.aspx (accessed on 9 April 2025). Population and demographic data were obtained from the General Authority for Statistics (GASTAT): https://www.stats.gov.sa/en/statistics-tabs.

## Abbreviations

The following abbreviations are used in this manuscript:

COVID-19	Coronavirus Disease 2019
EMR	Eastern Mediterranean Region
GASTAT	General Authority for Statistics (Saudi Arabia)
GCC	Gulf Cooperation Council
GHO	Global Health Observatory (WHO)
MCV1	First-dose measles-containing vaccine
MCV2	Second-dose measles-containing vaccine
MoH	Ministry of Health (Saudi Arabia)
RD	Rate difference
RR	Rate ratio
WHO	World Health Organization
WUENIC	WHO/UNICEF Estimates of National Immunization Coverage
